# NutriBrain: protocol for a randomised, double-blind, controlled trial to evaluate the effects of a nutritional product on brain integrity in preterm infants

**DOI:** 10.1186/s12887-021-02570-x

**Published:** 2021-03-17

**Authors:** Lisa M. Hortensius, Edith H. van den Hooven, Jeroen Dudink, Maria Luisa Tataranno, Ruurd M. van Elburg, Manon J. N. L. Benders

**Affiliations:** 1 Department of Neonatology, Wilhelmina Children’s Hospital, University Medical Center Utrecht, Utrecht University, Utrecht, The Netherlands; 2 University Medical Center Utrecht Brain Center, Utrecht University, Utrecht, The Netherlands; 3 Danone Nutricia Research, Utrecht, The Netherlands; 4 Emma Children’s Hospital, Amsterdam University Medical Center, University of Amsterdam, Amsterdam, The Netherlands

**Keywords:** Probiotics, Prebiotics, Glutamine, Preterm infants, Brain development, Randomised controlled trial

## Abstract

**Background:**

The gut microbiota and the brain are connected through different mechanisms. Bacterial colonisation of the gut plays a substantial role in normal brain development, providing opportunities for nutritional neuroprotective interventions that target the gut microbiome. Preterm infants are at risk for brain injury, especially white matter injury, mediated by inflammation and infection. Probiotics, prebiotics and L-glutamine are nutritional components that have individually already demonstrated beneficial effects in preterm infants, mostly by reducing infections or modulating the inflammatory response. The NutriBrain study aims to evaluate the benefits of a combination of probiotics, prebiotics and L-glutamine on white matter microstructure integrity (i.e., development of white matter tracts) at term equivalent age in very and extremely preterm born infants.

**Methods:**

This study is a double-blind, randomised, controlled, parallel-group, single-center study. Eighty-eight infants born between 24 + 0 and < 30 + 0 weeks gestational age and less than 72 h old will be randomised after parental informed consent to receive either active study product or placebo. Active study product consists of a combination of *Bifidobacterium breve *M-16V, short-chain galacto-oligosaccharides, long-chain fructo-oligosaccharides and L-glutamine and will be given enterally in addition to regular infant feeding from 48 to 72 h after birth until 36 weeks postmenstrual age. The primary study outcome of white matter microstructure integrity will be measured as fractional anisotropy, assessed using magnetic resonance diffusion tensor imaging at term equivalent age and analysed using Tract-Based Spatial Statistics. Secondary outcomes are white matter injury, brain tissue volumes and cortical morphology, serious neonatal infections, serum inflammatory markers and neurodevelopmental outcome.

**Discussion:**

This study will be the first to evaluate the effect of a combination of probiotics, prebiotics and L-glutamine on brain development in preterm infants. It may give new insights in the development and function of the gut microbiota and immune system in relation to brain development and provide a new, safe treatment possibility to improve brain development in the care for preterm infants.

**Trial registration:**

ISRCTN, ISRCTN96620855. Date assigned: 10/10/2017.

**Supplementary Information:**

The online version contains supplementary material available at 10.1186/s12887-021-02570-x.

## Background

In recent years a growing body of evidence has developed supporting the existence of a microbiome-gut-brain-axis [1–3]. The gut microbiota connect to the brain through different mechanisms that are not yet fully elucidated but likely consist of neural, immunological and endocrine pathways [4, 5]. Research has shown that bacterial colonisation of the gut is important for normal brain function making the gut microbiome a possible target for nutritional neuroprotective interventions. Studies in germ-free animals and animals exposed to bacterial infections suggest that the gut microbiota play a role in anxiety and behaviour [6, 7]. Interestingly, studies in mice have demonstrated beneficial effects of probiotics on brain function, mood and behaviour [6, 8], whilst studies in germ-free mice have established that altered behavioural responses could be normalised if normal gut microbiota was reconstituted early in life. Normalisation in adulthood failed to exert such an effect [9, 10]. These findings suggest a ‘window of opportunity’ early in life for the gut microbiota to influence brain development and behaviour that could be used in the care for preterm infants. Preterm infants are at risk for brain injury, especially white matter injury, because of their extra-uterine life during a period of significant brain development [11]. White matter injury, and also other brain injury, can cause disrupted development of the white matter and subsequent deteriorated white matter integrity. The impact of nutrition on brain growth and cognition in preterm infants is increasingly acknowledged, [12–17] although randomised controlled trials of many single nutritional components have demonstrated no clear positive effect of individual components on brain development of preterm infants so far [18]. For the current study, the active intervention will consist of three nutritional components combined: probiotics, prebiotics and glutamine. These three components have already demonstrated positive effects in preterm infants individually.

### Probiotics

Probiotics are living micro-organisms that in adequate amounts provide health benefits for the host, [19] using different mechanisms including alteration of the gut microbiota, increase in the gut epithelial barrier function and modulation of the immune system [20]. A number of clinical trials showed significant reduction in the incidence of severe necrotising enterocolitis (NEC), all-cause mortality and sepsis in preterm infants who received probiotics compared to controls [21–23]. Reduction of the risk of NEC and all-cause mortality, but not sepsis, in infants supplemented with probiotics compared to placebo was confirmed in a Cochrane review evaluating 24 studies and over 5000 infants [24]. A second systematic review and meta-analysis including 25 studies and over 6000 infants did find a reduction of the risk of sepsis in preterm infants who received probiotics compared to placebo, although many included studies were of low methodological quality [25]. For our study the probiotic strain *Bifidobacterium breve* (*B. breve*) M-16V was selected. A large body of evidence from both animal studies and clinical trials in infants shows that administration of *B. breve* M-16V is associated with beneficial health effects including modulation of the gut microbiota, [26, 27] reduced levels of pathogenic bacteria, [27] reduced inflammatory reactions, [28, 29] and prevention of infection and NEC, [29, 30] summarised in a recent review [31].

### Prebiotics

Prebiotics are non-digestible food components that can modify gut microbiota by being selectively fermented by beneficial intestinal bacteria, such as *Bifidobacteria* [32, 33]. The combination of probiotics and prebiotics can therefore work synergistically [34]. For this study short-chain (sc) galacto-oligosaccharides (GOS) and long-chain (lc) fructo-oligosaccharides (FOS), two of the most widely studied prebiotics, will be used. Oligosaccharides represent an important component of human milk and, interestingly, especially of preterm human milk [35]. They have multiple beneficial qualities including an ability to alter the gut microbiota and immunomodulatory functions [32]. Because of their presumed health benefits prebiotic oligosaccharides are supplemented to preterm formula as part of routine care. Several studies evaluated the effect of adding oligosaccharides to preterm infant formula on growth, intestinal microbiota, gastrointestinal transport time, stool frequency and stool consistency [36–39]. Prebiotic supplementation resulted in increased *Bifidobacteria* counts [36] and fewer intestinal pathogens, [37] accelerated gastrointestinal transport, [38, 39] increased stool frequency, [36] and reduced stool viscosity [38] in infants compared to controls. Furthermore, a meta-analysis showed a positive effect of prebiotic supplementation in preterm infants on the incidence of sepsis, mortality, length of hospital stay and time to reach full enteral feeding, but not on the incidence of NEC [40]. Supplementation with prebiotics may also modify the systemic inflammatory response by improving the balance between pro- and anti-inflammatory cytokines, with one study reporting lower cytokine levels in infants fed prebiotic oligosaccharides as a supplement to human milk or preterm formula compared to the control group, shortly after the start of the intervention [41].

### L-glutamine

L-glutamine is considered an important fuel for rapidly dividing cells such as enterocytes and lymphocytes and plays a substantial role in maintaining functional integrity of the gut [42, 43]. Previous studies in preterm infants have shown a positive effect of supplementation with L-glutamine on gastrointestinal dysfunction, [44] severe neurologic sequelae, [44] serious neonatal infections, [45, 46] head circumference [47] and brain volumes [48]. The effect of parenteral and enteral supplementation with glutamine on mortality and morbidity in preterm infants was evaluated in a Cochrane review. Although no overall difference in mortality or morbidity was found, a subgroup meta-analysis including studies with enteral supplementation only (6 studies, 1095 infants) showed a lower incidence of invasive infection in the glutamine supplemented group compared to controls (RR 0.76, 95% CI 0.64–0.89, I^2^ = 56%) [49]. Although this result is based on subgroup analysis and should therefore be interpreted with caution, it emphasises the possible effect of L-glutamine on preventing inflammation and its subsequent potential as a neuroprotective agent. 

To date there is no literature on the combined effects of *B. breve* M-16V, scGOS/lcFOS and L-glutamine in preterm infants nor their impact on neonatal brain development. However a recent preclinical trial with preterm born piglets (*n* = 40) showed those supplemented with *B. breve* M-16V, scGOS/lcFOS and L-glutamine demonstrated increased cognitive performance as assessed in the T-maze compared to control piglets [50]. Additionally, on ex vivo diffusion imaging piglets in the intervention group showed increased maturation of the white matter fibre bundles and advanced grey matter microstructural maturational processes compared to control piglets [50]. We hypothesize that this combination of probiotics, prebiotics and L-glutamine may act synergistically and will therefore combine these components in this study. We hypothesize that scGOS/lcFOS will promote survival of *B. breve* M-16V and improve its effect on immunomodulation and intestinal integrity in addition to its expected intrinsic benefits. L-glutamine may further enhance these favourable effects. This combination may decrease serious neonatal infections and subsequently reduce white matter injury. This study aims to evaluate the benefits of *B. breve* M-16V, scGOS/lcFOS and L-glutamine on white matter microstructure integrity (i.e., development of white matter tracts) at term equivalent age in very and extremely preterm born infants.

## Methods

### Study design and study setting

The NutriBrain study is a double-blind, randomised, controlled, parallel-group, single-center study. The aim of this study is to investigate the effect of the test product versus control given to preterm infants born at 24 + 0 to < 30 + 0 weeks gestational age (GA) on white matter microstructure integrity measured as fractional anisotropy (FA) assessed using magnetic resonance diffusion tensor imaging (DTI) at term equivalent age (TEA) and analysed using Tract-Based Spatial Statistics (TBSS).

This study will be conducted at the University Medical Center (UMC) Utrecht and seven regional hospitals in The Netherlands (details can be found in the ISRCTN registry). Due to the GA of included infants (extremely and very preterm infants) the inclusion, randomisation and study start will take place at the level III neonatal intensive care unit of the UMC Utrecht, a large tertiary care hospital in The Netherlands. If the condition of the infant has improved to the extent that intensive care is no longer necessary, they can be transferred to a level II or level I neonatal unit at a participating regional hospital and will continue to be followed up (under supervision of the principal investigator). Each hospital to which an infant is transferred will be asked to provide information relating to the study parameters during the infant’s stay.

### Study population

Infants with a GA of 24 + 0 to < 30 + 0 weeks that are less than 72 h old are eligible for inclusion. A complete overview of inclusion and exclusion criteria is shown in Table 1.

**Table 1:** Overview of inclusion and exclusion criteria

**Inclusion criteria**	
1) Gestational age of 24 + 0 to < 30 + 0 weeks	
2) Less than 72 h old and the intention to receive the first administration of study product between 48 and 72 h after birth	
3) Written informed consent from custodial parent(s)	
**Exclusion criteria**	
1) Any relevant proven or suspected chromosomal anomaly, metabolic disorder, genetic syndrome or congenital central nervous system malformation	
2) Presence of any gastrointestinal malformation	
3) No realistic prospect of survival (e.g. severe brain injury), at the discretion of the attending physician	
4) Concomitant participation in other intervention studies (for example, but not exclusively, those studies involving investigational or marketed nutritional or pharmaceutical products) that could impact on the main outcome parameters and/or infant safety	
5) Expected or foreseen inability of the infant and/or their families to adhere to protocol instructions	
6) Admission from an extra regional hospital, unless that hospital is a study site	
7) Current use of gastric acid inhibitors: H_2_-receptor antagonists (including ranitidine) or proton pump inhibitors (including omeprazole)	

### Treatment allocation and blinding

After eligibility has been assessed and informed consent has been obtained the infant will be enrolled in the study. To randomise an infant to the study, investigators will log in to the electronic Case Report Form (eCRF) and follow the prompts to input infant data. Only investigators specifically trained to perform randomisation will have access to the eCRF. Based on the order in which infants enter the study and stratified by sex, the infant will be assigned a randomisation number by the eCRF system. The infant will receive the product with the code corresponding to his/her randomisation number. Twins and multiple births will be allocated to the same study group. Investigators, all site staff including medical and nursing staff and supporting staff who prepare the infant feedings, and parents will be unaware of treatment allocation.

The randomisation sequence will be generated using the PLAN procedure of SAS statistical software (Enterprise Guide version 4.3 or higher) by a statistician from Danone Nutricia Research who has no further involvement in the conduct of the trial. The permuted block randomisation will be stratified by sex (boys, girls) with a 1:1 allocation ratio of test and control product. The block size or sizes and whether the block size(s) will be fixed or randomly varied will be decided by and known only to the statistician. The packaging of the test and control products will be identical in appearance. The study product for each infant will be labelled using a unique random number according to the randomisation schedule. These random numbers will have been linked (via a code) beforehand to either test or control product. Which numbers correspond to which treatment will only be known to the supplies manager at Danone Nutricia Research.

The randomisation code will be broken after analysis of the primary outcome and other outcomes collected until TEA. Assessors, parents, hospital staff and all those involved in the conduct and/or decision making of the follow-up phase will remain blinded to study product allocation until follow-up database lock after the last infant has completed the study (24 months corrected age). In the case of a medical emergency that requires preliminary unblinding of study product, unblinding in the eCRF or with sealed unblinding envelopes stored at the UMC Utrecht will be possible.

### Study products

The test product is a food for special medical purposes in powder form, used as a complement to the regular hospital feeding regimes of preterm infants. The product will be packaged into two separate sachets: Part A, containing *B. breve* M-16V, and Part B, containing prebiotic oligosaccharides scGOS and lcFOS in a ratio of 9:1, as well as L-glutamine. The control product will be similarly packaged into two separate sachets as follows: Part A, containing maltodextrin, and Part B, containing maltodextrin and casein and whey protein hydrolysates.

The study products will be provided to the study sites by Danone Nutricia Research and manufactured according to good manufacturing practice*.*

### Supplementation

Study product supplementation will start between 48 and 72 h after birth or, if the infant is clinically unstable as soon as possible thereafter, and will continue until 36 weeks postmenstrual age. If the infant is discharged home or fully breastfed before 36 weeks postmenstrual age supplementation will be stopped. Study product Part A will be administered enterally once per day as a single daily dose not supplemented to feeding or medication. The dosage of *B. breve* will depend on birth weight: Infants with a birth weight ≥ 1000 g will receive 3 × 10^9^ colony-forming units (cfu) per day; Infants with a birth weight < 1000 g will receive 1.5 × 10^9^ cfu per day, to be increased to 3 × 10^9^ cfu per day when they have reached enteral feeding intakes of 50 ml/kg/day. Study product Part B will be administered enterally supplemented to the regular milk feed (breast milk or preterm formula, depending on parents’ choice). Dosage will be increased in a stepwise fashion. Based on the infant’s weight, daily intake of enteral feeding and the desired (maximum) osmolarity of the feeding, a supplementation schema was created, starting with a minimum dosage of 0.05 g/day L-glutamine and 0.1 g/day scGOS/lcFOS and aiming for a final dose of 0.3 g/kg/day L-glutamine and 0.6 g/kg/day scGOS/lcFOS when the infant receives full enteral feeding (120 ml/kg/day for 1 day). The final dosages of L-glutamine and scGOS/lcFOS were based on the GEEF study [45] and CARROT study [51], respectively, in which supplementation was well tolerated.

### Outcome measures

#### Primary outcome

The primary outcome parameter in this study is integrity of white matter tracts throughout the brain, measured as FA assessed with DTI magnetic resonance imaging (MRI) at TEA. DTI detects the direction of diffusion of water molecules, which is influenced by the process of increasing myelination. Many studies have shown that FA - a measure of degree of diffusion in one direction - and tractography - a reconstruction of tracts using FA - are predictive of long-term neurodevelopmental outcome [52]. DTI images will be analysed using Tract-Based Spatial Statistics (TBSS), [53] part of the FMRIB Software Library [54]. TBSS is a voxelwise statistical method in which all infants FA data will be projected onto a mean FA tract skeleton and will be cross-subject analysed. TBSS analysis is sensitive as an imaging biomarker in the preterm population, [55] and can be used as a suitable biomarker of disease and treatment effects in children born very preterm [52, 55, 56].

#### Secondary outcomes

- White matter injury, measured with the white matter injury score according to Kidokoro et al., [57] which evaluates cystic lesions, focal signal abnormality, myelination delay, thinning of the corpus callosum, dilated lateral ventricles and volume reduction on T2- and T1-weighted MR images acquired at TEA.
- Brain tissue volumes (cerebellum, cortical grey matter, unmyelinated white matter, deep nuclear grey matter, ventricular volumes and extracerebral cerebrospinal fluid) and cortical morphology (sulcation index, cortical surface area and cortical thickness) assessed using the segmentation method of Moeskops et al. [58] of T2- and T1-weighted MR images made at TEA.
- The occurrence of serious neonatal infections (defined as culture proven or clinical sepsis with associated clinical symptoms; clinically significant NEC [i.e., Bell’s stage two or higher]; meningitis with or without positive culture; or pneumonia [positive culture of tracheal aspirate, bronchial secretion, or sputum positive for microorganisms with associated clinical symptoms]) [45, 51] until TEA.
- Serum concentrations of specific circulating inflammatory markers such as IL-6, IL-10, TNF-α and IL-8/CXCL8 measured with flow cytometry and Luminex at 7, 21 and 42 days postnatal, 30 and 36 weeks postmenstrual age, discharge to other hospital and TEA (optional, in a subgroup).
- Motor and cognitive development assessed with the Bayley Scales of Infant and Toddler Development, Third Edition, [59] at 24 months corrected age.

#### Safety outcomes

The following parameters will be assessed to evaluate safety of supplementation with the study product:

- Occurrence, type and duration of (Serious) Adverse Events.
- Adequate growth for weight (g/kg/week), length (cm/week) and head circumference (cm/week) until the end of intervention.
- Growth velocity and anthropometric z-scores until 24 months corrected age.
- Number of days of parenteral nutrition.
- Time in days to achieve full enteral nutrition (defined as 120 ml/kg/day for 1 day).
- Feeding tolerance (frequency and daily volume of gastric residuals, the occurrence of diarrhoea and constipation and stool frequency).
- Serum glutamine and glutamate concentrations (optional, in a subgroup).

### Study assessments

Table 2 (see Additional file 1) shows a timeline of all assessments for each infant.

### Data collection

All study data will be collected in a validated eCRF (VIEDOC, from Pharma Consulting Group during the intervention period; Castor during the follow up period). To guarantee data quality only trained study staff can enter data in the eCRF. Data will be collected and stored in line with data privacy laws, and details on data management are documented in the data management plan. Collected data will include: gender, GA, (birth) weight, (birth) length, (birth) head circumference, parental level of education, parental head circumference, score for Neonatal Acute Physiology – Perinatal Extension (SNAPPE-II), obstetric and perinatal details (such as presence of chorioamnionitis, prenatal medication, relevant maternal illnesses, Apgar scores and cord blood parameters), neonatal morbidity (such as infant respiratory distress syndrome, need for respiratory support, neonatal infections, patent ductus arteriosus, intraventricular haemorrhage and post haemorrhagic ventricular dilatation), neonatal medication, enteral and parenteral feeding characteristics and cumulative intake. Additionally stool samples will be collected at fixed time points to evaluate bacterial colonisation and the development of the microbiome and to be able to detect possible cross contamination with *B. breve* M-16V in infants allocated to the control group, using *B. breve* M-16V specific primers.

### Protocol compliance and missing data

Study product preparation and supplementation will be recorded in a log and the infant’s medical file and is therefore traceable. All study procedures will take place during the regular hospital admission or regular follow up. Only the MRI in a subgroup of infants (infants born between 28 + 0 and < 30 + 0 weeks GA with a birth weight ≥ 1000 g and with no significant abnormalities on serial cranial ultrasound images) will be specifically performed for study purposes, which will be clearly communicated with parents before informed consent is given. This will likely minimise non-compliance and missing data since the percentage of infants attending regular follow up in our hospital is very high (> 90%).

### Safety reporting

During the entire study period from first intake of study product until last follow up visit data on safety (i.e., Adverse Events and Serious Adverse Events) will be collected. An overview of Serious Adverse Events will be presented to the ethics committee regularly.

An independent Data Monitoring Committee (DMC) has been established to perform safety surveillance and to protect the scientific validity and credibility of the study. The DMC will make recommendations regarding safety and/or tolerance parameters and/or modifications in protocol based on the results of the safety evaluation, interim analyses and emerging evidence from other studies and/or relevant other sources of evidence. The DMC may also give the recommendation on continuation, termination or continuation with modifications for the study. The activities and responsibilities of the DMC have been planned following the recommendations of the DAMOCLES study related to the DMC members [60].

Two interim analyses will be performed by an independent statistician: one when 20 infants have completed the study until TEA and another when 40 infants evaluable for the interim analysis have completed the study until TEA. The objectives of the interim analyses are to evaluate safety and/or tolerance outcomes. Data for safety evaluation purposes and the interim analyses results will be supplied in strict confidence to the DMC. The safety evaluation and interim analyses will be conducted on semi-blinded data (“X” and “Y”). At all times the DMC has the right to decide to fully unblind the study groups or to unblind the treatment for one specific infant (e.g., in case of sepsis). As unique randomisation numbers will be used unblinding an individual infant will not lead to unblinding of others. In case of a clinically relevant (to be judged by the DMC) difference in death, sepsis, meningitis or NEC between the test group and control groups the DMC can recommend termination of the study. Unless decided otherwise by the DMC the study will be stopped if an infant is diagnosed with sepsis (a Serious Adverse Event) and *B. breve* M-16V is subsequently detected in the infant’s blood. In addition the DMC can recommend termination of the study for other reasons.

### Sample size calculation

The sample size needed for TBSS analyses has been estimated based on previous simulations of the sensitivity of TBSS for detecting treatment effects, as reported by Ball et al., who recruited infants with GA < 36 weeks who underwent successful DTI at TEA (baseline characteristics: median GA [range] 28 + 6 [23 + 4–35 + 2], median birthweight [range] 1130 g [630–3710 g]) [55]. The likelihood of TBSS detecting an effect of the intervention on the FA will depend on the overall FA increase and its distribution around the mean FA increase. The effect of the current intervention on FA is unknown. We hypothesize that the increase in FA in the intervention group will be moderate, in the order of 5%. TBSS analyses the relation between different parameters (e.g., GA, sex, intervention group) and the FA of voxels of the white matter skeleton (i.e., the inter-subject aligned centre of white matter tracts). Based on the simulations in neonates reported by Ball et al., in order to detect an estimated increase in FA of 5% in approximately 50% of the TBSS skeletal voxels 35 infants per intervention group are required [55]. They showed that TBSS can detect clinically relevant global differences in FA, even in small groups and can therefore be used as a powerful biomarker of white matter development [55]. In a recent study comparing the effects of high vs. lower breast milk exposure in 20 vs. 27 preterm infants group wise differences in brain connectivity at TEA were shown with TBSS [61]. Based on these results we assume this whole-brain DTI analysis technique is sensitive to the effects of our intervention with 35 evaluable infants per intervention group. In total 70 evaluable infants will be needed. To account for potential drop-outs and non-evaluable infants, the target population should consist of 88 randomised infants. This number includes allocated twins/multiple births.

### Statistical analysis

#### Primary outcome parameter and other MRI-derived parameters

TBSS analysis will be performed as previously described [61]. In summary, group comparisons will be performed with FMRIB Software Library’s Randomise tool using a general linear univariate model, [62] with family-wise error correction for multiple testing using threshold-free cluster enhancement with a significance level of *p* < 0.05 [63]. Group comparisons for volumetric analyses will be performed with ANOVA, with false discovery rate to correct for multiple testing. In all analyses GA at birth, postmenstrual age at time of MRI, birth weight Z-score and days of ventilation will be entered as covariates. These analyses have been used in several other observational and intervention studies conducted in the UMC Utrecht and other research groups, in preterm infants at TEA (mean and median GA of 28.7 weeks), [52, 64] healthy and asphyxiated full term infants, [65, 66] as well as children at school age (7.5–8.5 years and 12 years of age) [67–69].

#### Safety and tolerance parameters, exploratory outcome parameters and secondary outcome parameters that are not derived from MRI assessment

Statistical analysis of continuous outcomes that are measured at more than one time point will be performed by means of linear mixed models analysis. Statistical analysis of discrete outcomes (dichotomous variables, ordered categorical variables and counts) will be performed using parametric (i.e., generalized linear models) or non-parametric methods. For other types of outcome parameters stratified tests (e.g., Cochran-Mantel-Haenszel test) and/or simple hypothesis testing methods (e.g., Chi-square or Fisher exact tests) will be employed. Details on the statistical analyses will be documented in a statistical analyses plan prior to database lock/unblinding.

Considering our relatively small sample size, it is possible that confounding factors for probiotic efficacy, such as antibiotic exposure, could be unevenly distributed between the active and placebo study group. Baseline characteristics will be evaluated at the start of the statistical analysis, and important confounding factors will be taken into account as covariates in the analysis. Analysis of the primary outcome parameter will be based on all included patients intended to treat with usable MRI according to DTI quality. Additionally, a per protocol analysis will be carried out for supporting purposes. Safety parameters will be analysed on the All Subjects Treated population. To account for possible cross contamination additional analyses will be performed to evaluate infants with and without *B. breve* M-16V colonisation. A *p*-value < 0.05 will be considered statistically significant.

## Discussion

Infection and inflammation, together with ischemia, play an important role in white matter injury. These processes cause upregulation of pro-inflammatory cytokines and activate microglia. Together this leads to excitotoxicity and free radical attack, causing cell death of premyelinating oligodendrocytes and subsequent glial scarring of the white matter [70]. It is therefore likely that reduction of infectious morbidity may have a positive effect on brain integrity through less disturbance of white matter structure. Multiple possible mechanisms contribute to the proposed effect of probiotics, prebiotics and L-glutamine on infectious morbidity. The bacterial colonisation of the gastrointestinal tract affects the immune system through several pathways [71] including the production of immunomodulatory factors such as short chain fatty acids [72]. Production of short chain fatty acids by intestinal bacteria is essential for numerous brain functions, such as microglia maturation and function [73] and maintaining integrity of the blood-brain-barrier [74]. Another postulated mechanism is one of improved white matter integrity through direct interaction with immune cells. *B. breve* attenuates the pro-inflammatory response in mouse macrophages in vitro [75]. As described previously probiotics, prebiotics and L-glutamine regulate bacterial colonisation, improve intestinal mucosal integrity and decrease bacterial translocation. This shifts the immune system to a more anti-inflammatory phenotype, indeed enteral supplementation of prebiotic fibers has been shown to decrease systemic cytokine levels (IL-1β, IFN-γ and TNF-α) in preterm infants at day 7 of life [41].

An important, well-known factor in microbiome development is the frequency and duration of antibiotic treatment. Preterm infants are regularly exposed to antibiotics for suspected infection. As spontaneous preterm birth itself could be an expression of infection antibiotic treatment is routinely started directly after birth in very and extremely preterm infants. Furthermore late onset sepsis (starting > 72 h after birth) is a common problem in very and extremely preterm infants, leading to additional antibiotic exposure. Even though the aim of antibiotic treatment is to diminish pathogenic bacteria beneficial bacteria are also affected, causing an altered microbiome development [76, 77]. Multiple studies have shown that (prolonged) antibiotic exposure leads to a less diverse microbiome [78–82]. We expect that the effect of our intervention will be influenced by exposure to antibiotics since courses of antibiotics will most likely diminish the growth of *B. breve*. We will include infants only in one hospital in which the incidence of late onset sepsis in extremely low birth weight infants (birth weight ≤ 1000 g) is approximately 27% [83]. Based on the previous intervention studies with probiotics, prebiotics and glutamine separately the intervention may also reduce the incidence of (suspected) infections, which may reduce the antibiotic exposure [25, 45, 51]. It is possible that the results of our study could be different in other hospitals with other antibiotic policies.

Our study will concern a very vulnerable, critically ill study population and the safety of the infants will be monitored very closely. Based on the results from previous studies with probiotics, prebiotics and L-glutamine as individual supplements no major side effects related to the study products are expected. The only recognised possible adverse reaction associated with probiotic administration is a positive culture of the probiotic organism from a normally sterile site. This is very rare and has never been reported with *B. breve* M-16V supplementation. For prebiotic supplementation in general mild gastrointestinal discomfort (loose stools, constipation, abdominal pain and flatulence) has been reported. However, in a recent systematic review and meta-analysis no safety concerns were raised from 18 evaluated randomised controlled trials on prebiotic supplementation in preterm infants [40]. No adverse events related to L-glutamine supplementation in preterm infants have been described [44–46, 48, 84]. For both prebiotic and L-glutamine supplementation the dosages that will be used in our study are equal to or below the dosages used in the previously mentioned studies.

This trial focusses on the effect of the active study product on brain development. Several important clinical outcomes, such as occurrence of serious infections, growth and neurodevelopmental outcome, are secondary or safety outcomes of this trial. We hope that our trial can serve as a first step in the development of future studies in which the effect of our active study product on clinical outcomes can be evaluated. To date we are not able to completely prevent perinatal brain injury in preterm infants. To our knowledge the NutriBrain study is the first to evaluate the effect of a combination of probiotics, prebiotics and L-glutamine on brain development in preterm infants. It may give new insights in the development and function of the gut microbiota and immune system in relation to brain development and may provide a new, safe treatment possibility with few potential side effects, to improve brain development in the care for preterm infants.

## Supplementary Information

**Additional file 1.** Timeline for maximum number of assessments for each infant. Table according to the SPIRIT guidelines that provides an overview of all study assessments for each infant.

## Data Availability

Not applicable.

## Abbreviations

*B. breve*: *Bifidobacterium breve*
cfu: colony-forming unit
DMC: Data monitoring committee
DTI: Diffusion tensor imaging
eCRF: electronic case report form
FA: Fractional anisotropy
GA: Gestational age
lcFOS: long-chain fructo-oligosaccharides
MRI: Magnetic resonance imaging
NEC: Necrotizing enterocolitis
scGOS: short-chain galacto-oligosaccharides
TBSS: Tract-based spatial statistics
TEA: Term equivalent age
UMC: University medical center

## References

[1] Cryan JF, O'Mahony SM (2011). The microbiome-gut-brain axis: from bowel to behavior. Neurogastroenterol Motil.

[2] Bercik P, Collins SM, Verdu EF (2012). Microbes and the gut-brain axis. Neurogastroenterol Motil.

[3] Mayer EA (2011). Gut feelings: the emerging biology of gut-brain communication. Nat Rev Neurosci.

[4] Cryan JF, Dinan TG (2012). Mind-altering microorganisms: the impact of the gut microbiota on brain and behaviour. Nat Rev Neurosci.

[5] Keunen K, van Elburg RM, van Bel F, Benders MJ (2015). Impact of nutrition on brain development and its neuroprotective implications following preterm birth. Pediatr Res.

[6] Gareau MG, Wine E, Rodrigues DM, Cho JH, Whary MT, Philpott DJ (2011). Bacterial infection causes stress-induced memory dysfunction in mice. Gut..

[7] Neufeld KM, Kang N, Bienenstock J, Foster JA (2011). Reduced anxiety-like behavior and central neurochemical change in germ-free mice. Neurogastroenterol Motil.

[8] Bravo JA, Forsythe P, Chew MV, Escaravage E, Savignac HM, Dinan TG (2011). Ingestion of Lactobacillus strain regulates emotional behavior and central GABA receptor expression in a mouse via the vagus nerve. Proc Natl Acad Sci U S A.

[9] Diaz Heijtz R, Wang S, Anuar F, Qian Y, Bjorkholm B, Samuelsson A (2011). Normal gut microbiota modulates brain development and behavior. Proc Natl Acad Sci U S A.

[10] Sudo N, Chida Y, Aiba Y, Sonoda J, Oyama N, Yu XN (2004). Postnatal microbial colonization programs the hypothalamic-pituitary-adrenal system for stress response in mice. J Physiol.

[11] Volpe JJ (2009). Brain injury in premature infants: a complex amalgam of destructive and developmental disturbances. Lancet Neurol.

[12] Ehrenkranz RA, Das A, Wrage LA, Poindexter BB, Higgins RD, Stoll BJ (2011). Early nutrition mediates the influence of severity of illness on extremely LBW infants. Pediatr Res.

[13] Ehrenkranz RA, Dusick AM, Vohr BR, Wright LL, Wrage LA, Poole WK (2006). Growth in the neonatal intensive care unit influences neurodevelopmental and growth outcomes of extremely low birth weight infants. Pediatrics..

[14] Franz AR, Pohlandt F, Bode H, Mihatsch WA, Sander S, Kron M (2009). Intrauterine, early neonatal, and postdischarge growth and neurodevelopmental outcome at 5.4 years in extremely preterm infants after intensive neonatal nutritional support. Pediatrics..

[15] Isaacs EB, Gadian DG, Sabatini S, Chong WK, Quinn BT, Fischl BR (2008). The effect of early human diet on caudate volumes and IQ. Pediatr Res.

[16] Isaacs EB, Morley R, Lucas A (2009). Early diet and general cognitive outcome at adolescence in children born at or below 30 weeks gestation. J Pediatr.

[17] Vinall J, Grunau RE, Brant R, Chau V, Poskitt KJ, Synnes AR (2013). Slower postnatal growth is associated with delayed cerebral cortical maturation in preterm newborns. Sci Transl Med.

[18] Hortensius LM, van Elburg RM, Nijboer CH, Benders M, de Theije CGM (2019). Postnatal nutrition to improve brain development in the preterm infant: a systematic review from bench to bedside. Front Physiol.

[19] Food and Agriculture Organization of the United Nations and World Health Organization. Health and Nutrition Properties of Probiotics in Food including Powder Milk with Live Lactic Acid Bacteria. Report of a Joint FAO/WHO Expert Consultation on Evaluation of Health and Nutritional Properties of Probiotics in Food including Powder Milk with Live Lactic Acid Bacteria. Cordoba, Argentina, 1–4 October 2001.

[20] Bermudez-Brito M, Plaza-Diaz J, Munoz-Quezada S, Gomez-Llorente C, Gil A (2012). Probiotic mechanisms of action. Ann Nutr Metab.

[21] Bin-Nun A, Bromiker R, Wilschanski M, Kaplan M, Rudensky B, Caplan M (2005). Oral probiotics prevent necrotizing enterocolitis in very low birth weight neonates. J Pediatr.

[22] Lin HC, Hsu CH, Chen HL, Chung MY, Hsu JF, Lien RI (2008). Oral probiotics prevent necrotizing enterocolitis in very low birth weight preterm infants: a multicenter, randomized, controlled trial. Pediatrics..

[23] Manzoni P, Rinaldi M, Cattani S, Pugni L, Romeo MG, Messner H (2009). Bovine lactoferrin supplementation for prevention of late-onset sepsis in very low-birth-weight neonates: a randomized trial. JAMA..

[24] AlFaleh K, Anabrees J (2014). Probiotics for prevention of necrotizing enterocolitis in preterm infants. Cochrane Database Syst Rev.

[25] Zhang GQ, Hu HJ, Liu CY, Shakya S, Li ZY (2016). Probiotics for preventing late-onset Sepsis in preterm neonates: a PRISMA-compliant systematic review and meta-analysis of randomized controlled trials. Medicine (Baltimore).

[26] Patole S, Keil AD, Chang A, Nathan E, Doherty D, Simmer K (2014). Effect of Bifidobacterium breve M-16V supplementation on fecal bifidobacteria in preterm neonates--a randomised double blind placebo controlled trial. Plos One.

[27] Li Y, Shimizu T, Hosaka A, Kaneko N, Ohtsuka Y, Yamashiro Y (2004). Effects of bifidobacterium breve supplementation on intestinal flora of low birth weight infants. Pediatr Int.

[28] Izumi H, Minegishi M, Sato Y, Shimizu T, Sekine K, Takase M (2015). Bifidobacterium breve alters immune function and ameliorates DSS-induced inflammation in weanling rats. Pediatr Res.

[29] Satoh T, Izumi H, Iwabuchi N, Odamaki T, Namba K, Abe F (2016). Bifidobacterium breve prevents necrotising enterocolitis by suppressing inflammatory responses in a preterm rat model. Benef Microbes.

[30] Yamashiro Y, Nagata S (2010). Beneficial microbes for premature infants, and children with malignancy undergoing chemotherapy. Benef Microbes..

[31] Wong CB, et al. Exploring the Science behind Bifidobacterium breve M-16V in Infant Health. Nutrients. Nutrients. 2019;11(8):1724. 10.3390/nu11081724 PMC6723912 31349739

[32] Davani-Davari D, et al. Prebiotics: Definition, Types, Sources, Mechanisms, and Clinical Applications. Foods. 2019;8(3):92. 10.3390/foods8030092 PMC6463098 30857316

[33] Gibson GR, Scott KP, Rastall RA, Tuohy KM, Hotchkiss A, Dubert-Ferrandon A (2010). Dietary prebiotics: current status and new definition. Food Sci Tech Bull Funct Foods.

[34] Srinivasjois R, Rao S, Patole S (2013). Prebiotic supplementation in preterm neonates: updated systematic review and meta-analysis of randomised controlled trials. Clin Nutr.

[35] Gabrielli O, Zampini L, Galeazzi T, Padella L, Santoro L, Peila C (2011). Preterm milk oligosaccharides during the first month of lactation. Pediatrics..

[36] Boehm G, Lidestri M, Casetta P, Jelinek J, Negretti F, Stahl B (2002). Supplementation of a bovine milk formula with an oligosaccharide mixture increases counts of faecal bifidobacteria in preterm infants. Arch Dis Child Fetal Neonatal Ed.

[37] Kapiki A, Costalos C, Oikonomidou C, Triantafyllidou A, Loukatou E, Pertrohilou V (2007). The effect of a fructo-oligosaccharide supplemented formula on gut flora of preterm infants. Early Hum Dev.

[38] Mihatsch WA, Hoegel J, Pohlandt F (2006). Prebiotic oligosaccharides reduce stool viscosity and accelerate gastrointestinal transport in preterm infants. Acta Paediatr.

[39] Indrio F, Riezzo G, Raimondi F, Francavilla R, Montagna O, Valenzano ML (2009). Prebiotics improve gastric motility and gastric electrical activity in preterm newborns. J Pediatr Gastroenterol Nutr.

[40] Chi C, Buys N, Li C, Sun J, Yin C (2019). Effects of prebiotics on sepsis, necrotizing enterocolitis, mortality, feeding intolerance, time to full enteral feeding, length of hospital stay, and stool frequency in preterm infants: a meta-analysis. Eur J Clin Nutr.

[41] van den Berg JP, van Zwieteren N, Westerbeek EA, Garssen J, van Elburg RM (2013). Neonatal modulation of serum cytokine profiles by a specific mixture of anti-inflammatory neutral and acidic oligosaccharides in preterm infants. Cytokine..

[42] Young VR, Ajami AM (2001). Glutamine: the emperor or his clothes?. J Nutr.

[43] Neu J, DeMarco V, Li N (2002). Glutamine: clinical applications and mechanisms of action. Curr Opin Clin Nutr Metab Care.

[44] Vaughn P, Thomas P, Clark R, Neu J (2003). Enteral glutamine supplementation and morbidity in low birth weight infants. J Pediatr.

[45] van den Berg A, van Elburg RM, Westerbeek EA, Twisk JW, Fetter WP (2005). Glutamine-enriched enteral nutrition in very-low-birth-weight infants and effects on feeding tolerance and infectious morbidity: a randomized controlled trial. Am J Clin Nutr.

[46] Sevastiadou S, Malamitsi-Puchner A, Costalos C, Skouroliakou M, Briana DD, Antsaklis A (2011). The impact of oral glutamine supplementation on the intestinal permeability and incidence of necrotizing enterocolitis/septicemia in premature neonates. J Matern Fetal Neonatal Med.

[47] de Kieviet JF, Vuijk PJ, van den Berg A, Lafeber HN, Oosterlaan J, van Elburg RM (2014). Glutamine effects on brain growth in very preterm children in the first year of life. Clin Nutr.

[48] de Kieviet JF, Oosterlaan J, Vermeulen RJ, Pouwels PJ, Lafeber HN, van Elburg RM (2012). Effects of glutamine on brain development in very preterm children at school age. Pediatrics..

[49] Moe-Byrne T, Brown JV, McGuire W (2016). Glutamine supplementation to prevent morbidity and mortality in preterm infants. Cochrane Database Syst Rev.

[50] Andersen AD, Nguyen DN, Langhorn L, Renes IB, van Elburg RM, Hartog A (2019). Synbiotics combined with glutamine stimulate brain development and the immune system in preterm pigs. J Nutr.

[51] Westerbeek EA, van den Berg JP, Lafeber HN, Fetter WP, Boehm G, Twisk JW (2010). Neutral and acidic oligosaccharides in preterm infants: a randomized, double-blind, placebo-controlled trial. Am J Clin Nutr.

[52] van Kooij BJ, de Vries LS, Ball G, van Haastert IC, Benders MJ, Groenendaal F (2012). Neonatal tract-based spatial statistics findings and outcome in preterm infants. AJNR Am J Neuroradiol.

[53] Smith SM, Jenkinson M, Johansen-Berg H, Rueckert D, Nichols TE, Mackay CE (2006). Tract-based spatial statistics: voxelwise analysis of multi-subject diffusion data. Neuroimage..

[54] Smith SM, Jenkinson M, Woolrich MW, Beckmann CF, Behrens TE, Johansen-Berg H (2004). Advances in functional and structural MR image analysis and implementation as FSL. Neuroimage..

[55] Ball G, Boardman JP, Arichi T, Merchant N, Rueckert D, Edwards AD (2013). Testing the sensitivity of tract-based spatial statistics to simulated treatment effects in preterm neonates. Plos One.

[56] Ceschin R, Lee VK, Schmithorst V, Panigrahy A (2015). Regional vulnerability of longitudinal cortical association connectivity: associated with structural network topology alterations in preterm children with cerebral palsy. Neuroimage Clin.

[57] Kidokoro H, Neil JJ, Inder TE (2013). New MR imaging assessment tool to define brain abnormalities in very preterm infants at term. AJNR Am J Neuroradiol.

[58] Moeskops P, Viergever MA, Mendrik AM, de Vries LS, Benders MJ, Isgum I (2016). Automatic segmentation of MR brain images with a convolutional neural network. IEEE Trans Med Imaging.

[59] Bayley N (2006). Bayley scales of infant and toddler development 3rd edition (Bayley-III).

[60] Damocles Study Group (2005). A proposed charter for clinical trial data monitoring committees: helping them to do their job well. Lancet..

[61] Blesa M, Sullivan G, Anblagan D, Telford EJ, Quigley AJ, Sparrow SA (2019). Early breast milk exposure modifies brain connectivity in preterm infants. Neuroimage..

[62] Winkler AM, Ridgway GR, Webster MA, Smith SM, Nichols TE (2014). Permutation inference for the general linear model. Neuroimage..

[63] Smith SM, Nichols TE (2009). Threshold-free cluster enhancement: addressing problems of smoothing, threshold dependence and localisation in cluster inference. Neuroimage..

[64] Ball G, Counsell SJ, Anjari M, Merchant N, Arichi T, Doria V (2010). An optimised tract-based spatial statistics protocol for neonates: applications to prematurity and chronic lung disease. Neuroimage..

[65] Feng K, Rowell AC, Andres A, Bellando BJ, Lou X, Glasier CM (2019). Diffusion tensor MRI of white matter of healthy full-term newborns: relationship to neurodevelopmental outcomes. Radiology..

[66] Porter EJ, Counsell SJ, Edwards AD, Allsop J, Azzopardi D (2010). Tract-based spatial statistics of magnetic resonance images to assess disease and treatment effects in perinatal asphyxial encephalopathy. Pediatr Res.

[67] Ou X, Andres A, Cleves MA, Pivik RT, Snow JH, Ding Z (2014). Sex-specific association between infant diet and white matter integrity in 8-y-old children. Pediatr Res.

[68] Samara A, Feng K, Pivik RT, Jarratt KP, Badger TM, Ou X (2019). White matter microstructure correlates with memory performance in healthy children: a diffusion tensor imaging study. J Neuroimaging.

[69] Jamieson D, Broadhouse KM, McLoughlin LT, Schwenn P, Parker MJ, Lagopoulos J (2020). Investigating the association between sleep quality and diffusion-derived structural integrity of white matter in early adolescence. J Adolesc.

[70] Khwaja O, Volpe JJ (2008). Pathogenesis of cerebral white matter injury of prematurity. Arch Dis Child Fetal Neonatal Ed.

[71] Hooper LV, Littman DR, Macpherson AJ (2012). Interactions between the microbiota and the immune system. Science..

[72] Dalile B, Van Oudenhove L, Vervliet B, Verbeke K (2019). The role of short-chain fatty acids in microbiota-gut-brain communication. Nat Rev Gastroenterol Hepatol.

[73] Erny D (2015). Hrabe de Angelis AL, Jaitin D, Wieghofer P, Staszewski O, David E, et al. host microbiota constantly control maturation and function of microglia in the CNS. Nat Neurosci.

[74] Silva YP, Bernardi A, Frozza RL (2020). The Role of Short-Chain Fatty Acids From Gut Microbiota in Gut-Brain Communication. Front Endocrinol (Lausanne).

[75] Okada Y, Tsuzuki Y, Hokari R, Komoto S, Kurihara C, Kawaguchi A (2009). Anti-inflammatory effects of the genus Bifidobacterium on macrophages by modification of phospho-I kappaB and SOCS gene expression. Int J Exp Pathol.

[76] Westerbeek EA, van den Berg A, Lafeber HN, Knol J, Fetter WP, van Elburg RM (2006). The intestinal bacterial colonisation in preterm infants: a review of the literature. Clin Nutr.

[77] Henderickx JGE, Zwittink RD, van Lingen RA, Knol J, Belzer C (2019). The preterm gut microbiota: an inconspicuous challenge in nutritional neonatal care. Front Cell Infect Microbiol.

[78] Gasparrini AJ, Wang B, Sun X, Kennedy EA, Hernandez-Leyva A, Ndao IM (2019). Persistent metagenomic signatures of early-life hospitalization and antibiotic treatment in the infant gut microbiota and resistome. Nat Microbiol.

[79] Gibson MK, Wang B, Ahmadi S, Burnham CA, Tarr PI, Warner BB (2016). Developmental dynamics of the preterm infant gut microbiota and antibiotic resistome. Nat Microbiol.

[80] Dardas M, Gill SR, Grier A, Pryhuber GS, Gill AL, Lee YH (2014). The impact of postnatal antibiotics on the preterm intestinal microbiome. Pediatr Res.

[81] Fouhy F, Guinane CM, Hussey S, Wall R, Ryan CA, Dempsey EM (2012). High-throughput sequencing reveals the incomplete, short-term recovery of infant gut microbiota following parenteral antibiotic treatment with ampicillin and gentamicin. Antimicrob Agents Chemother.

[82] Greenwood C, Morrow AL, Lagomarcino AJ, Altaye M, Taft DH, Yu Z (2014). Early empiric antibiotic use in preterm infants is associated with lower bacterial diversity and higher relative abundance of Enterobacter. J Pediatr.

[83] Ran NC, van den Hoogen A, Hemels MAC (2019). Gram-negative late-onset Sepsis in extremely low birth weight infants is emerging in the Netherlands despite quality improvement programs and antibiotic stewardship!. Pediatr Infect Dis J.

[84] Mercier A, Eurin D, Poulet-Young V, Marret S, Dechelotte P (2003). Effect of enteral supplementation with glutamine on mesenteric blood flow in premature neonates. Clin Nutr.

