# Supplementary material for: NutriBrain: protocol for a randomised, double-blind, controlled trial to evaluate the effects of a nutritional product on brain integrity in preterm infants
Source: BMC Pediatr. 2021 Mar 17;21:132. doi: 10.1186/s12887-021-02570-x (PMC7968155; doi:10.1186/s12887-021-02570-x)
Supplement: Supplementary file 1 — Additional file 1. Timeline for maximum number of assessments for each infant. Table according to the SPIRIT guidelines that provides an overview of all study assessments for each infant. [file 12887_2021_2570_MOESM1_ESM.docx]

Table 2: Timeline for maximum number of assessments for each infant.

|  |  | Screening, baseline & randomisation | Intervention | | | | | | | | Follow-up | | | |
| --- | --- | --- | --- | --- | --- | --- | --- | --- | --- | --- | --- | --- | --- | --- |
|  | Time point  -1 | Time point 0  Screening & Baseline | Time point 1  Start Study | Time point 2 | Time point 3 | Time point 4 | Time point 5 | Time point 6 | Time point 7  End of intervention | | Time point 8 Primary outcome parameter | Follow-up visit 1  Time point 9 | Follow-up visit 2  Time point 10 | Follow-up visit 3  Time point 11  End of Study |
|  | Antenatally to 24 hours after birth | 0-72 hours after birth | As soon as possible (from 48-72 hours after birth) | 7 days postnatal | 21 days postnatal | 42 days postnatal | 30 weeks PMA (29+5-32+0 weeks) | Discharge other hospital | 36 weeks PMA^#^ | | TEA (40+0-42+0 weeks) | 4 months CA | 15 months CA | 24 months CA |
|  | -1 week-  day 1 | day 1-3 postnatal | Day 2-3 postnatal | 1-week | 3-weeks | 6-weeks | 2-6 weeks | 1-16 weeks* | 6-12 weeks | | 11-17 weeks | 6-8 months | 17-19 months | 26-28 months |
| Informed consent | (x) | x |  |  |  |  |  |  |  | |  |  |  |  |
| In/exclusion criteria | x |  |  |  |  |  |  |  |  | |  |  |  |  |
| Randomisation |  | x |  |  |  |  |  |  |  | |  |  |  |  |
| Study product intake |  |  | From 48-72 hours after birth until 36 weeks PMA^#^ | | | | | | | |  |  |  |  |
| Demographic, obstetric, perinatal details |  | x |  |  |  |  |  |  |  | |  |  |  |  |
| Medical history |  | x |  |  |  |  |  |  |  | |  |  |  |  |
| Neonatal morbidity |  |  | x | x | x | x | x | x | x | | x |  |  |  |
| Anthropometric data |  | x | x | x | x | x | x | x | x | | x | x | x | x |
| Feeding details |  | x | x | x | x | x | x | x | x | |  | x | x | x |
| Skin, oral cavity, and nasopharynx swab |  |  |  | x | x | x |  | x | x | | x | x | x | x |
| Blood sample for biomarkers |  |  |  | x | x | x | x | x | x | | x |  |  |  |
| Stool sample for physiology/Microbiota |  | x |  | x | x | x |  | x | x | | x | x | x | x |
| MRI |  |  |  |  |  |  | x |  |  | | x |  |  |  |
| Questionnaire for allergy, infection, and number of doctor visits |  |  |  |  |  |  |  |  |  | |  | x | x | x |
| BSITD-III |  |  |  |  |  |  |  |  |  | |  |  |  | x |
| (S)AEs |  | | Recorded from first intake of the study product onwards | | | | | | | | | | | |
| Concomitant medication |  | | Recorded throughout the intervention period and follow up period | | | | | | | | | | | |
| Nutritional supplements |  | | Recorded throughout the intervention period | | | | | | |  | | | | |

Legend:

Number of assessments depends on infant’s gestational age, age at transfer to regional hospital and type of regional hospital.

PMA: postmenstrual age; TEA: term equivalent age; CA: corrected age; MRI: magnetic resonance imaging; BSITD-III: Bayley Scales of Infant and Toddler Development, third edition; (S)AE: (serious) adverse event.

* Not a fixed time point, depending on the infant’s clinical condition.

^#^ Or discharge home, or fully breastfed, whichever comes first.
